# Lipidomics Combined with Network Pharmacology to Explore Differences in the Mechanisms of Grey Hair Development Between Type 2 Diabetes Mellitus and Normal Populations (Female)

**DOI:** 10.3390/ijms26052034

**Published:** 2025-02-26

**Authors:** Liwen Wu, Shiqi Li, Congfen He

**Affiliations:** 1 Department of Cosmetics, Beijing Technology and Business University, Beijing 100048, China; 2 Shanghai Skin Disease Hospital, School of Medicine, Tongji University, Shanghai 200443, China

**Keywords:** type 2 diabetes mellitus, grey hairs, lipidomics, UPLC-QTOF-MS, OPLS-DA, network pharmacology

## Abstract

Type 2 diabetes is usually accompanied by premature grey hair. In this study, we analysed differences in the lipid composition of black and white hair follicles between women with type 2 diabetes and healthy populations, using lipidomic methods. We examined the correlation between the lipid composition of female grey hair follicles and type 2 diabetes mellitus, and we screened for potential grey-hair-delaying ingredients using network pharmacology. Forty-one female volunteers with type 2 diabetes (diabetes, D) and thirty-five healthy volunteers (healthy, H) aged 55–65 years were recruited. Hair roots, including the follicular portion, were collected from grey hair (D-W for diabetic volunteers and H-W for healthy volunteers) and black hair (D-B for diabetic volunteers and H-B for healthy volunteer). Lipids were extracted separately and analysed using UPLC-QTOF-MS (Ultra-Performance Liquid Chromatography–Tandem Time-of-Flight Mass Spectrometry), combined with an OPLS-DA (Orthogonal Partial Least Squares Discriminant Analysis) model to identify different lipids among different groups under VIP conditions (VIP > 1, *p* < 0.05, and fold change ≥ 2). Further screening was performed using the ROC (receiver operating characteristic) curve method, selecting lipids with an AUC (area under the curve) value greater than 0.8 and specificity plus sensitivity greater than 1.6. Finally, bioinformatics and reverse network pharmacology were used to screen relevant targets, ingredients, and herbs to find suitable raw materials with anti-grey-hair effects. We found the following: (1) Ten significant differential lipids were identified under VIP conditions in the D-W and D-B groups, and five potential differential lipids (1-O-alpha-D-glucopyranosyl-1,2-eicosandiol, emmotin A, odyssic acid, PI-Cer(t18:0/26:0(2OH)), and NAPE(18:1(9Z)/16:1(9Z)/18:0)) were further screened using ROC analysis. The levels of all five lipids were significantly higher in D-W than in D-B, and these elevated levels may have been related to the production of grey hair in diabetic patients. (2) Thirteen significantly different lipids were screened under VIP conditions in the H-W and H-B groups, and five potential differential lipids were screened via ROC analysis (PS(O-16:0/13:0), PA(12:0/16:1(9Z)), PS(13:0/20:3(8Z,11Z,14Z)), GlcCer(d18:1/24:1(15Z)), and PS(O-20:0/17:2(9Z,12Z))). The levels of all five lipids were significantly higher in H-B than in H-W, and we hypothesised that their reduced levels were associated with the production of grey hair in the healthy population. (3) Twelve significantly different lipids were screened under VIP conditions in the D-W and H-W groups, and two potential differential lipids were screened via ROC analysis (fucoxanthinol 3-heptadecanoate 3′-myristate and 2-(3-hydroxyphytanyl)-3-phytanyl-sn-glycerol). The contents of both lipids were significantly higher in H-W than in D-W, and there were differences in the lipid composition of grey hair in the D and H populations. (4) Important ingredients with possible therapeutic effects were obtained through lipid-matched target screening: resveratrol, calycosin, epigallocatechin 3-gallate, and herbs such as the fruit of the glossy privet, etc. In summary, the production of grey hair in the D and H populations may be affected by different lipids. The lipid components emmotin A and fucoxanthinol 3-heptadecanoate 3′-myristate were significantly higher in the D and H populations than in the same groups (D-B, H-B), and these are pregnenolone lipids (PRs). We hypothesised that PRs can influence the production of grey hair in both populations. The screening of important differential lipids may serve to provide diagnostic loci or therapeutic targets, while matching ingredients and herbs may provide a basis and direction for the subsequent development of anti-grey-hair ingredients.

## 1. Introduction

Dysregulation in the balance of the melanocyte spectrum within the hair follicle is the underlying cause of physiological and pathological grey hair [1]. During the progressive deterioration of cellular functions with ageing, melanin synthesis is impeded after tyrosinase activity is reduced [2]. In addition, genotoxic stress in the hair bulge reduces the number of melanin stem cells, while oxidative stress in the hair bulb leads to melanin stem cell dysfunction [3]. The loss of melanin stem cells’ ability to move between hair follicle growth zones can cause grey hair [4]. In addition to physiological grey hair, diabetic patients may also experience premature grey hair as a result of their disease. According to traditional Chinese medicine, type 2 diabetes mainly affects the kidneys, with Yin deficiency and dry heat being the mechanisms underlying its occurrence, development, and resolution [5]. In Chinese medicine, the *Zhu Bing Yuan Hou Lun* treatise describes how deficiencies in the body’s qi and blood weaken the kidneys, leading to further bone marrow depletion and, ultimately, the hair turning grey. A fraction of follicular lipids play a role in regulating hair growth and pigment production [6]. One study has highlighted the importance of lipid modification in grey hair, revealing that brown and white hair follicles have different lipid contents at the root. Specifically, phospholipids, vitamin D3, and cholesterol are significantly lower in white hair follicles [7]. Decreased lipid content across all parts of the hair shaft—the stratum corneum, cortex, and medulla—with age also affects the clinical characteristics of grey hair [8]. Not many studies have been conducted on the role of lipids in hair, and most research on the correlation between diabetes mellitus and grey hair has focused mainly on genes [9], with few studies on the lipid-related mechanisms of grey hair development in type 2 diabetes mellitus.

Lipidomics analysis, a systematic process analysis, elucidates the mechanisms of lipids’ action in physiological changes in the body by comparing the total amounts of small-molecule metabolites in lipids in different physiological and pathological states in the body. This form of analysis can identify important lipid biochemical indices in metabolic regulation. Our research group has established a complete set of lipid collection, extraction, data collection, and analysis methods based on UPLC-QTOF-MS (Ultra-Performance Liquid Chromatography–Tandem Time-of-Flight Mass Spectrometry). The effects of lipids on symptom characteristics are demonstrated through lipid identification and component analysis. In this study, the participants were divided into a diabetic white-hair group (D-W), a diabetic black-hair group (D-B), a healthy white-hair group (H-W), and a healthy black-hair group (H-B). The lipidomics analysis method was used to systematically and comprehensively analyse lipids in the black and white hair follicles of the type 2 diabetic women and compare them with those of a healthy population. We did so to compare the composition of hair follicle lipids in the black and white hair of women with type 2 diabetes mellitus.

OPLS-DA (Orthogonal Partial Least Squares Discriminant Analysis) is a multivariate statistical analysis method that is mainly used to deal with classification problems in high-dimensional data (e.g., metabolomics and transcriptomics data). VIP values filter out variables that are not meaningful for categorisation in multivariate models. Variables with VIP > 1 contribute significantly more to categorical models than random noise, a key feature in distinguishing differences between groups. The *p*-value verifies the statistical significance of a variable and compensates for the risk of possible overfitting of a VIP value; *p* < 0.05 indicates that the probability that the between-group difference between the variables is due to random error is less than 5%. A fold change ≥ 2 is usually considered to indicate a potential change in biological function. The same threshold was used in a study on facial lipids [10]. Therefore, these conditions were chosen as thresholds for the initial screening of lipids in this study.

The ROC (receiver operating characteristic) curve is an important tool for evaluating the performance of a binary classification model. It quantifies the classification ability of the model by calculating the area under the curve (AUC). The ROC curve—with specificity on the horizontal scale and sensitivity on the vertical scale—demonstrates the false positive rate (FPR) and true positive rate (TPR) at different thresholds. An AUC > 0.8 is usually considered to indicate a good model performance. In this study, AUC > 0.8 and specificity plus sensitivity > 1.6 were used to screen the lipids [11].

Network pharmacology is an emerging field that combines biology, bioinformatics, network science, and other disciplines [12]. Reverse network pharmacology builds on known disease and gene targets of action to reverse-backtrack potential ingredients or drugs that may act on intersecting targets [13]. Using white-hair-related lipids obtained via lipidomics analysis, we used reverse network pharmacology to screen the effective raw materials for preventing white hair.

## 2. Results

### 2.1. Differences in Lipids Between D-B and D-W Hair Follicles

#### 2.1.1. Results of Lipid Separation Between D-B and D-W Hair Follicles

Sample collection was performed in positive ion mode for groups D-B and D-W. After collection, the data were entered into Progenesis QI V2.0 to complete peak extraction. Ezinfo 3.0 was used in OPLS-DA mode, and the resulting data were imported to obtain score plots and observe the differences in lipid composition between the two groups.

**Figure 1:**
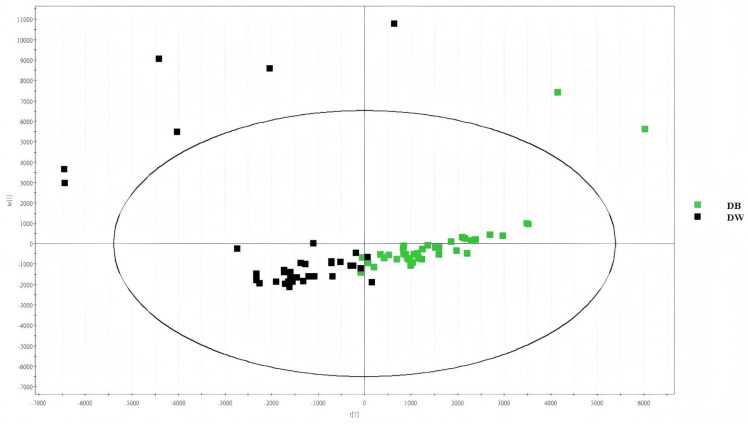
OPLS-DA score graphs for D-B and D-W.

By calculating the gap between the OPLS-DA and the pattern recognition evaluation groups, we obtained a score map. The horizontal coordinates of the OPLS-DA score map represent the scoring values of the main components, and the direction of the horizontal coordinates shows the gap between the groups. The scores of the orthogonal components are represented by the vertical coordinates; the differences within the groups can also be seen in these coordinates (the same is shown below). As shown in Figure 1, the degree of lipid separation between the two groups was good, indicating that the difference between the two was large. Each point in Figure 1 represents a sample. The diabetic black-hair group (D-B) is represented by green, and the diabetic white-hair group (D-W) is represented by black. Figure 1 shows that the D-W and D-B lipids were separated, indicating that their information in this part of the sample differed. Thus, we can further analyse these lipids.

#### 2.1.2. Lipid Screening Results Between D-B and D-W Hair Follicles

**Figure 2:**
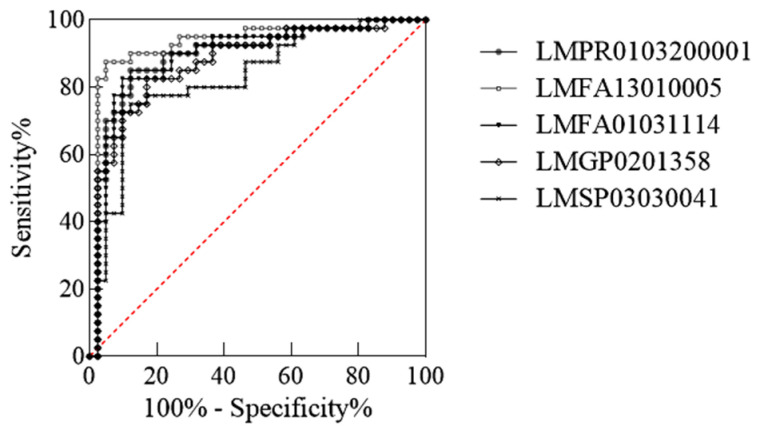
ROC curves of potential differential lipids in D-B and D-W.

**Figure 3:**
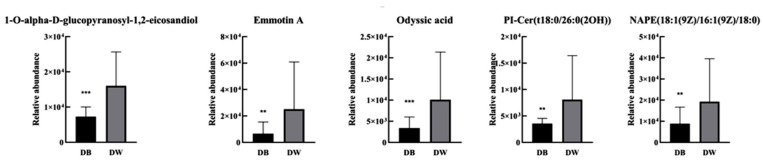
Potential differential lipid contents between D-B and D-W (*** *p* < 0.01; ** *p* < 0.1).

With *p* < 0.05, fold change ≥ 2, and VIP > 1 as the screening conditions, we found 10 lipids with significant differences between D-B and D-W (Table 1). The 1-O-alpha-D-glucopyranosyl-1,2-icosandiol content in fatty acids (FAs) was the most different between these lipids, followed by emmotin A in pregnenolone lipids (PRs). The abundance of these 10 different lipids was analysed using the ROC method. Five potentially differentiated lipids with AUC values greater than 0.8 and specificity plus sensitivity greater than 1.6 were screened (Figure 2), all of which were present at higher levels in grey hair than in black hair (Figure 3).

### 2.2. Differences in Lipids Between H-B and H-W Hair Follicles

#### 2.2.1. Results of Lipid Separation Between H-B and H-W Hair Follicles

**Figure 4:**
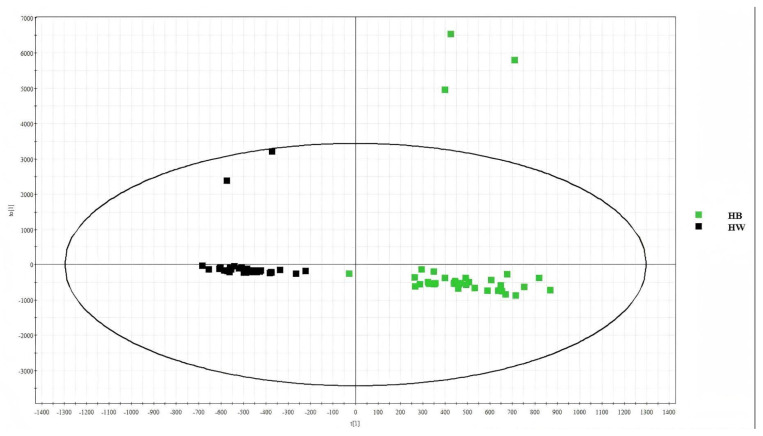
OPLS-DA score graphs for H-B and H-W.

The samples were collected and analysed in the same way as described in Section 2.1.1. In the OPLS-DA score map for H-B and H-W, the healthy black-hair group is represented by green, and the healthy white-hair group is represented by black. Figure 4 shows that lipids in the H-W and H-B groups were separated, and their information in this part of the sample differed. Thus, the lipids could be further analysed.

#### 2.2.2. Lipid Screening Results Between H-B and H-W Hair Follicles

**Figure 5:**
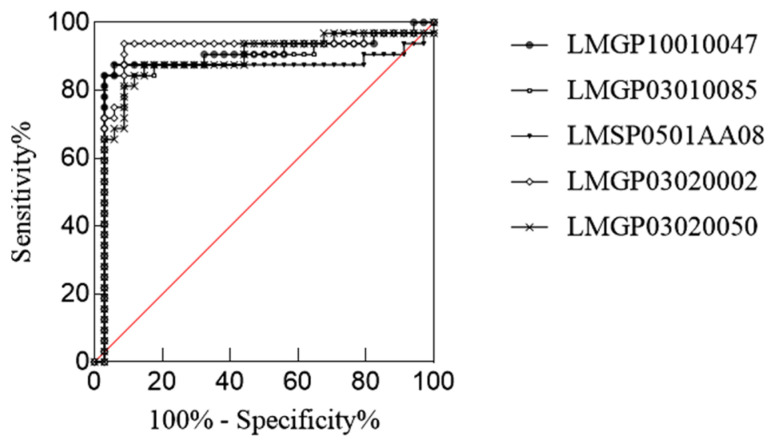
ROC curves of potential differential lipids in H-B and H-W.

**Figure 6:**
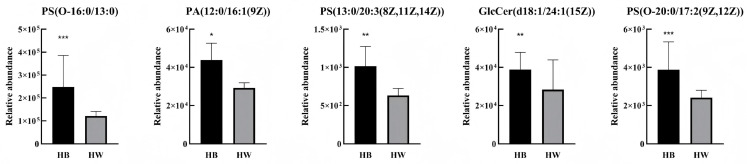
Potential differential lipid contents between H-B and H-W (*** *p* < 0.001, ** *p* < 0.01, and * *p* < 0.5).

With *p* < 0.05, fold change ≥ 2, and VIP > 1 as the screening conditions, we found 13 lipids with significant differences between the H-B and H-W groups (Table 2). PS(O-16:0/13:0) had the greatest difference in lipid content, followed by PA(12:0/16:1(9Z)), both of which belong to the glycerophospholipid group (GP). The amounts of these 13 different lipids were analysed using the ROC method. Five potentially differentiated lipids with AUC values greater than 0.8 and specificity plus sensitivity greater than 1.6 were screened (Figure 5), all of which were present at higher levels in black hair than in grey hair (Figure 6).

By comparing the D-B and D-W groups with the H-B and H-W groups, respectively, we found that the lipids in the black and grey hairs were significantly different between the two groups, indicating that different lipids affect the production of these kinds of hair differently in diabetic and healthy people. NAPE(18:1(9Z)/16:1(9Z)/18:0) in group D (diabetes group), and PS(O-16:0/13:0), PS(13:0/20:3(8Z,11Z,14Z)), PS(O-20:0/17:2(9Z,12Z)) and PA(12:0/16:1(9Z)) in group H (healthy group) belong to the GP. PI-Cer(t18:0/26:0(2OH)) in group D and Glc-Cer(d18:1/24:1(15Z)) in group H are sphingolipids (SPs). However, according to the experimental results, the NAPE(18:1(9Z)/16:1(9Z)/18:0) and PI-Cer(t18:0/26:0(2OH)) contents in D-W were significantly higher than those in D-B, and the PS(O-16:0/13:0), PS(13:0/20:3(8Z,11Z,14Z)), PS(O-20:0/17:2(9Z,12Z)), PA(12:0/16:1(9Z)), and Glc-Cer(d18:1/24:1(15Z)) contents in H-B were significantly higher than those in H-W. This indicates that the effects of similar lipids on black and white hair in diabetic patients and healthy people are different. Therefore, we suspect that there is a difference in lipid composition between the white hair of diabetic patients and healthy people.

### 2.3. Differences in Lipids Between D-W and H-W Hair Follicles

#### 2.3.1. Results of Lipid Separation Between D-W and H-W Hair Follicles

**Figure 7:**
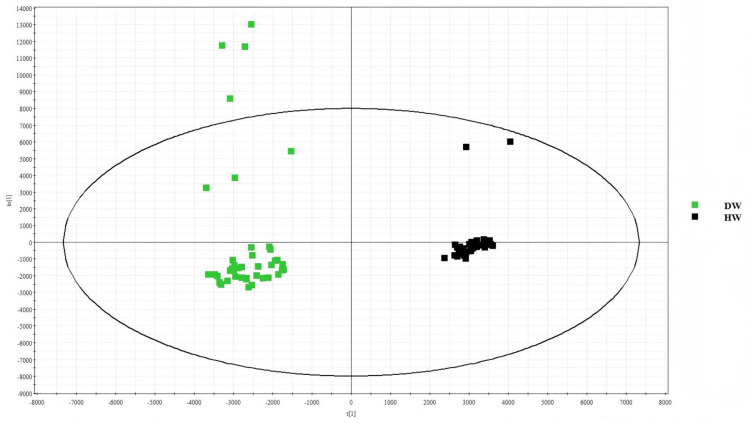
OPLS-DA score graphs for D-W and H-W.

Samples were collected and analysed in the same way as described in Section 2.1.1. In the OPLS-DA score diagram for D-W and H-W, the diabetic white-hair group is represented by green, and the diabetic black-hair group is represented by black. Figure 7 shows that there is a certain degree of separation between D-W and H-W, and the lipid information in this part of the sample differs. Thus, the lipids can be further analysed.

#### 2.3.2. Lipid Screening Results Between D-W and H-W Hair Follicles

**Figure 8:**
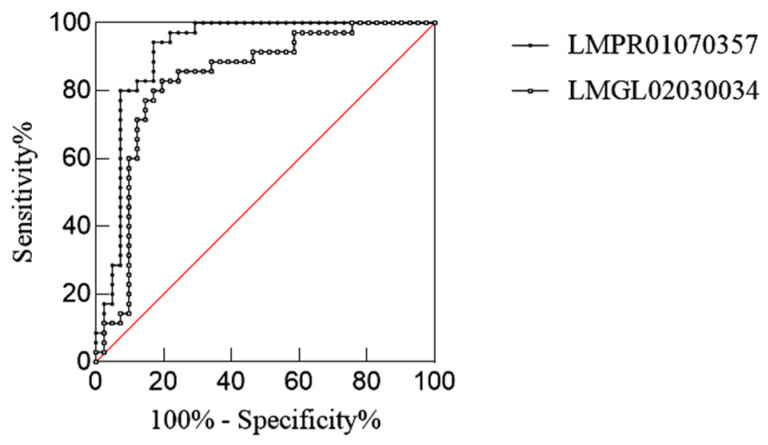
ROC curves of potential differential lipids in D-W and H-W.

**Figure 9:**
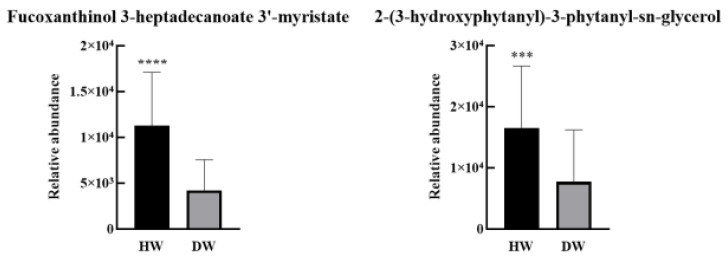
Potential differential lipid contents between D-W and H-W (**** *p* < 0.0001; *** *p* < 0.001).

With *p* < 0.05, fold change ≥ 2, and VIP > 1 as the screening conditions, we found 12 lipids with significant differences in D-W and H-W (Table 3). Fucoxanthinol 3-heptadecanoate 3′-myristate in pregnenolone lipids (PRs) showed the greatest differences between these groups. The amounts of these 12 lipids were analysed using the ROC method. Two potentially differentiated lipids with AUC values greater than 0.8 and specificity plus sensitivity greater than 1.6 were screened (Figure 8), both of which were present at significantly higher levels in the healthy group than in the diabetic group (Figure 9).

### 2.4. Ingredient Screening of the Differential Lipids Emmotin A and Fucoxanthinol 3-Heptadecanoate 3-Myristate

#### 2.4.1. Relevant Target Screening and Gene Interaction Network Analysis

In a previous study, the hair roots of volunteers in groups D and H were studied using untargeted lipidomics. the results showed that two different lipids of pr—emmotin a and 3-heptadecanoate 3′-myristate fluoxanthinol—were associated with grey hair production. a total of 489 relevant targets were retrieved from pharmmapper (http://www.lilab-ecust.cn/pharmmapper/, accessed on 10 November 2023), a platform for pharmacokinetic matching and potential target identification, and 308 targets were obtained after removing duplicates.

**Figure 10:**
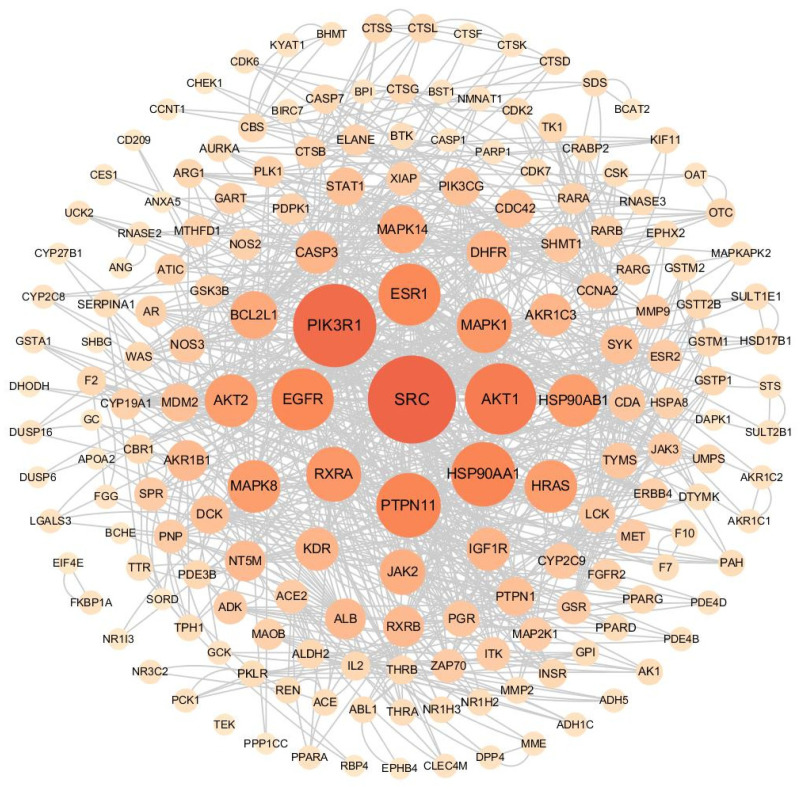
Network diagram of gene interactions.

The 308 targets were uploaded to the String12.0 network database to create a gene interaction network graph and obtain the corresponding data. A total of 306 nodes were obtained, with an average node degree value of 3.2. The filtering conditions were adjusted to the minimum interaction threshold, “highest confidence > 0.9”, hiding the nodes with broken networks. We exported the data as tabular text and imported them into Cytoscape 3.7.1 for more intuitive network graph construction and to calculate degree values. In Figure 10, darker colours and larger nodes indicate that the gene interacts more strongly with other genes. The top nine genes in terms of degree values were *SRC* (degree, 58), *PIK3R1* (degree, 54), *AKT1* (degree, 44), *PTPN11* (degree, 38), *HSP90AA1* (degree, 38), *ESR1* (degree, 36), *EGFR* (degree, 36), *RXRA* (degree, 30), and *MAPK1* (degree, 30).

#### 2.4.2. Screening Ingredients and Herbs

**Figure 11:**
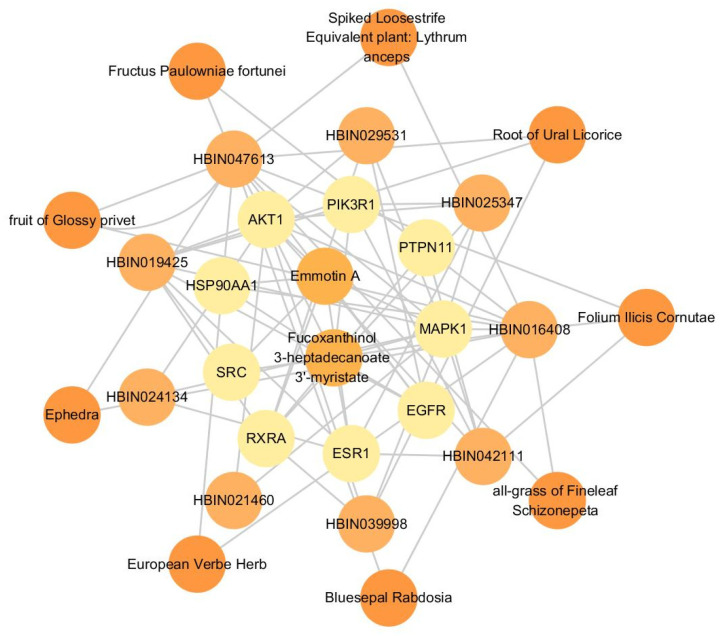
Lipids–top nine targets–top nine ingredients–top nine herbs network diagram.

The top nine targets in terms of degree values were entered into HERB (http://herb.ac.cn/, accessed on 10 November 2023) and matched to 988 different ingredients. The degree value of each ingredient in the “lipid–target–ingredient” network was calculated using Cytoscape 3.7.1. The top nine ingredients are shown in Table 4. Furthermore, 442 herbs were matched in HERB using these nine ingredients. A network of the “lipid–top nine targets–top nine ingredients–top nine herbs” (Figure 11) was constructed using Cytoscape 3.7.1, and the herbs (Table 5) were verified in the literature.

The fruit of the glossy privet can cause hair acne by increasing TYR activity, enhancing melanin-synthesis-related gene expression and melanin production [14]. Alcoholic extracts of this fruit can promote the growth of human scalp hair follicles cultured in vitro, as well as the expression of melanocyte c-kit receptor proteins and melanocyte TYR mRNA, which can increase melanin production [15]. The monomer tyrosol and oleanolic acid in glossy privet fruit can cause hair acne by enhancing the expression of the related protein TRP-1, increasing TYR activity, and affecting the biological activity of melanocytes [16,17].

#### 2.4.3. Pathway Enrichment Analysis of Targets

**Figure 12:**
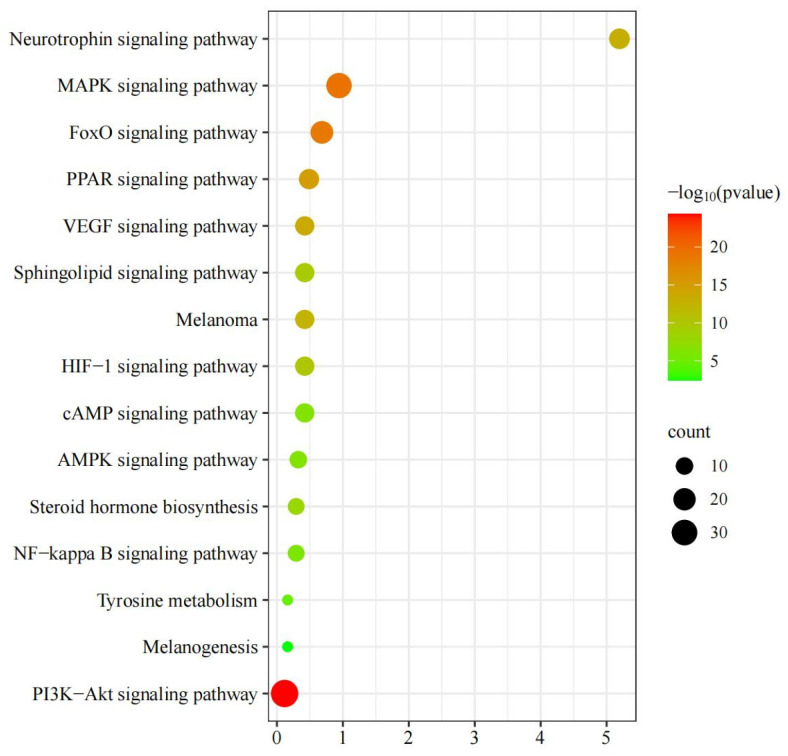
KEGG enrichment analysis.

**Figure 13:**
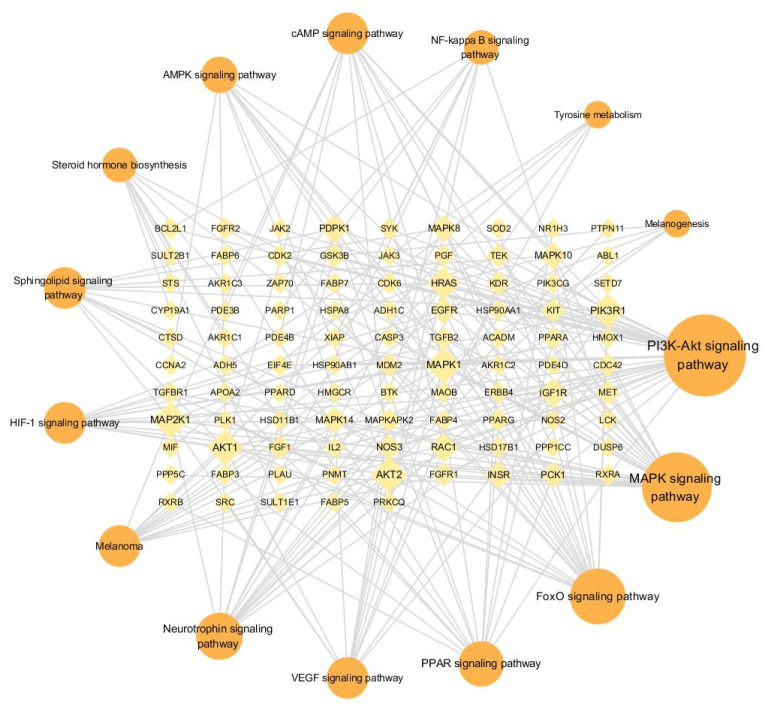
KEGG pathway–gene network diagram.

The 308 gene column names obtained from pharmmapper—a platform for pharmacophore matching and potential target identification—were imported into Metascape (https://metascape.org, accessed on 15 November 2023), a functional annotation bioinformatics analysis platform. The input species and analysed species were limited to humans (“*H. sapiens*”) and then subjected to personalised analysis (“Custom Analysis”). The enrichment conditions were set as follows—min overlap: 3; *p*-value cutoff: 0.01; min enrichment: 1.5. A total of 202 pathways were obtained from the enrichment analysis of the 308 targets using the “KEGG pathway”. Of these, 15 pathways were related to hair growth or melanin synthesis (Figure 12 and Table 6); a pathway–gene network diagram is shown in Figure 13. The top nine genes were *AKT1* (degree, 10), *AKT2* (degree, 10), *MAPK1* (degree, 10), *MAP2K1* (degree, 10), *PIK3R1* (degree, 9), *HRAS* (degree, 8), *IGF1R* (degree, 6), *PDPK1* (degree, 6), and *RAC1* (degree, 6).

#### 2.4.4. Verification of Important Active Ingredients via Molecular Docking

**Figure 14:**
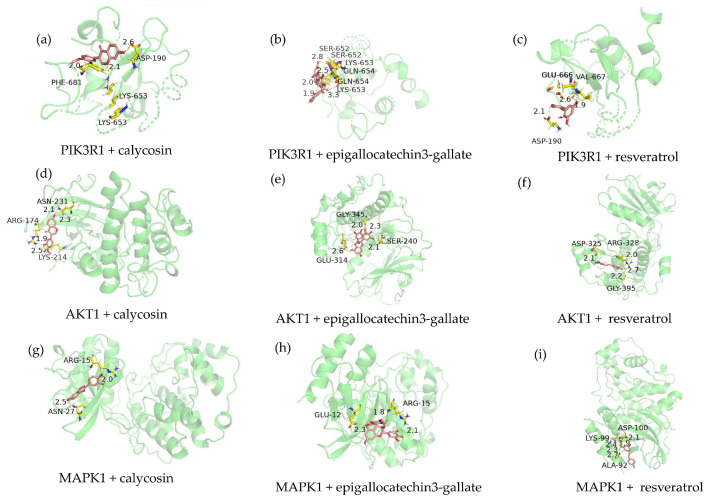
Ingredient–target docking conformations (green: target protein; pink: ingredient; yellow: amino acid residues; red and blue for the system automatically assigned, no special meaning).

The genes in the gene interaction network and the KEGG pathway–gene network were sorted from high to low according to degree value. The top nine gene names of both were intersected to obtain the *PIK3R1*, *AKT1*, and *MAPK1* genes, which were referred to as important genes for the occurrence of white hair. The top three ingredients were resveratrol, calycosin, and epigallocatechin 3-gallate. The structures of these three important ingredients were obtained through the organic small-molecule biological activity database PubChem (https://pubchem.ncbi.nlm.nih.gov/, accessed on 21 November 2023). Using the open babel 3.1.1 software, an sdf-format compound structure file was transformed into a pdbqt-format file. Important protein targets were saved in the pdbqt format after removing water molecules and excess ions and adding hydrogen ions using the AutoDockTools 4 software. The molecular docking of the important protein targets and the important active ingredients was performed using AutoDockTools 4. The molecular docking binding energy scores were obtained, as shown in Table 7, and the molecular docking results were visualised using PyMOL 2.5.5 (Figure 14).

## 3. Discussion

This study compared the differences in hair follicle lipids between women with type 2 diabetes and healthy women with black and grey hair. We screened for possible differences in lipids based on VIP conditions and using the ROC method, as well as for ingredients and herbs that may be effective in delaying grey hair via reverse network pharmacology. The main results were as follows: (1) The differential lipids in groups D-B and D-W were 1-O-alpha-D-glucopyranosyl-1,2-eicosandiol, emmotin A, odyssic acid, PI-Cer(t18:0/26:0(2OH)), and NAPE(18:1(9Z)/16:1(9Z)/18:0). (2) The differential lipids in groups H-B and H-W were PS(O-16:0/13:0), PA(12:0/16:1(9Z)), PS(13:0/20:3 (8Z,11Z,14Z)), GlcCer(d18:1/24:1(15Z)), and PS(O-20:0/17:2(9Z,12Z)). (3) The differential lipids in groups D-W and H-W were fucoxanthinol 3-heptadecanoate 3′-myristate and 2-(3-hydroxyphytanyl)-3-phytanyl-sn-glycerol. (4) Ingredients that may be effective in delaying grey hair include resveratrol, calycosin, and epigallocatechin 3-gallate. Finally, a herb that may be effective in delaying grey hair is the glossy privet fruit.

Odyssic acid is an unsaturated fatty acid. Research [29] has shown that zebrafish V12RAS-driven melanoma can remove free fatty acids. Indeed, some overlaps between the altered lipid metabolism genes in the zebrafish melanoma model and human melanoma were emphasised in this study and showed clinical significance. The higher odyssic acid content in D-W may be related to this. 1-O-alpha-D-glucopyranosyl-1,2-eicosandiol was more abundant in D-W, but the intrinsic mechanism was not clear. This compound and odyssic acid both belong to the fatty acid class, so the cause of its higher content in white hair may be similar to that of odyssic acid. Emmotin A belongs to the sesquiterpenoid class. The literature shows that terpenoids have an inhibitory effect on tyrosinase activity [30], thus explaining the higher levels of emmotin A in grey hair than in black hair. One study [31] found that the PS content of follicular lipids in the hair of women with early grey hair was higher in grey hair than in black hair, presumably because of reduced melanin production due to the significant apoptosis of melanocytes in grey hair. NAPE belongs to the same glycerophospholipid class (GP) as PS, so the high NAPE content in D-W compared with black hair may also be related to melanocyte apoptosis. PI-Cer is a sphingolipid (SP) compound, and ceramide (Cer), a common sphingomyelin base derivative, is the structural parent of all sphingolipids, including sphingoglycolipids and sphingomyelins [32]. As some of the most important sphingolipid molecules, ceramides can mediate most sphingolipid biological functions. It has been hypothesised that Cer inhibits melanin synthesis by inhibiting extracellular signal-regulated protein kinase (ERK) and Akt/protein kinase B (PKB), delaying the activation of both [33]. This mechanism may also account for the higher levels of PI-Cer in grey hair.

Ageing weakens the antioxidant system, and the significant accumulation of reactive oxygen species causes the unsaturated bonds of lipids to be attacked by these species, significantly decreasing the amount of PS in the cell membrane [34]. This explains the lower amount of PS in H-W. PS belongs to the glycerophospholipid class, and it has been shown [29] that zebrafish V12RAS-driven melanocyte tumours can upregulate the production of certain glycerophospholipids. By reviewing the literature, we also know the following: PA’s function in animal cells mainly includes improving cell survival and promoting cell proliferation, the MAPK signalling pathway is important for hair melanogenesis [14], and the key step in activating this signalling pathway is the transfer of Raf from the cytoplasm to the cytosol and its activation by Ras or other kinases. The binding of Raf to PA can promote its transfer to the cytosol [35]. Patent EPWO0112141, a hair growth promoter, has PA as one of its main ingredients [36]; therefore, the PA content in black hair is higher than that in white hair. GlcCer in sphingolipids (SPs) is the precursor for the production of most GSLs, and the absence of GSLs causes tyrosinase to accumulate in the Golgi, leading to a reduced capacity for melanin synthesis [37]; therefore, there is more GlcCer in black hair.

Fucoxanthinol 3-heptadecanoate 3′-myristate belongs to the terpenoid class, and triterpenoids are used to regulate blood glucose and treat diabetes [38]. Thus, it has been hypothesised that fucoxanthinol 3-heptadecanoate 3′-myristate has a similar effect, resulting in lower levels of D-W than H-W. 2-(3-Hydroxyphytanyl)-3-phytanyl-sn-glycerol in glycerolipids (GLs) belongs to the glycerol diester group; it can lower blood glucose, improve insulin sensitivity, and slow the process of diabetes [39]; therefore, its content in D-W is much lower.

## 4. Materials and Methods

### 4.1. Reagents and Instruments

Lipids were collected using sebum sampling paper. They were extracted using reagents such as ammonium formate, acetonitrile, formic acid, methanol, isopropanol, chloroform, and acetone. Lipid composition analysis was performed using ACQUITY UPLC I Class/Xevo G2-XS Q-TOF (Waters, Milford, MA, USA).

### 4.2. Lipid Collection and Extraction

In total, 41 diabetic volunteers (female) and 35 healthy volunteers (female) aged 55–65 years were recruited for lipid collection. Our inclusion criteria for the participants were menopause and no other skin disorders. The exclusion criteria were pre-existing skin lesions or previous use of hormonal drugs. The diabetes grade was assessed by an endocrinologist using the China Guideline for Type 2 Diabetes. Informed consent was obtained from each participant before any samples were collected. The study was conducted in accordance with the Declaration of Helsinki, and it was approved by the Ethics Committee of the Beijing University Shougang Hospital (SHERLL2019030).

Three black and white hairs with follicular lipids were plucked from diabetic volunteers and healthy volunteers. The parts with follicular lipids were taped to separate sebum sampling papers, and the excess hairs were cut off. The tape samples were folded and placed in labelled centrifuge tubes, which were immediately stored on dry ice. After completing all of the sampling work on the same day, all samples were placed in a refrigerator at −80 °C for retention.

After removing the samples from the refrigerator at −80 °C, we added a mixture of methanol and chloroform, waited for 3 min, and then added an equal volume of acetone solution. Following this, the tape was picked off after 10 min. The centrifuge tube was blown with a nitrogen blower until there was no liquid in the tube; then, a mixture of methanol and isopropanol was added, and the supernatant was centrifuged and injected into an inserted tube.

### 4.3. Liquid and Mass Spectrometry Conditions

The chromatographic column used in this experiment was a UPLC CSH C18 (Waters, Milford, MA, USA); the mobile phases were phase A (water and acetonitrile, 3:2 *v*/*v*, mixed homogeneously, with ammonium formate content of 10 mmol/L and formic acid content of 0.1%) and phase B (isopropanol and acetonitrile, 1:9 *v*/*v*, mixed homogeneously, with ammonium formate content of 10 mmol/L and formic acid content of 0.1%). The total injection volume was 2.0 μL, the flow rate was 400 μL/min, and the column temperature was 60 °C. The gradient elution procedure for liquid chromatography was as follows: at 0~1 min, the ratio of phases A and B was 6:4; at 16 min, only the B phase was left, without the A phase; at 18 min, only the B phase was left, without the A phase; at 20.1~22 min, the ratio of phases A and B was 6:4.

Lipids were acquired using an electrospray (ESI) ion source in positive ionisation mode, which was controlled over a mass scan range of 50–1200 *m*/*z*, with nitrogen vented into each gas channel. To determine the mass accurately, leucine enkephalin (*m*/*z* 554.2771) was chosen as the external standard for this experiment. Data acquisition was performed using the MassLynx 4.1 data collation system.

Partial mass spectrometry parameters: the temperature of the solvent gas was 400 °C, the voltage of the capillary was 3200 V(+)/2500 V(−), the temperature of the ion source was 120 °C, the flow rate of the solvent gas was 800 L/H, and the voltage of the conical hole was 35 V.

### 4.4. Lipid Data Collection and Analysis Methods

Initial data for lipid analysis were acquired using the MassLynx 4.1 management software, and they were subsequently processed using Ezinfo 3.0 and Progenesis QI V2.0. The specific steps were as follows: The initial data were imported from the QI software, and the peaks were extracted and aligned. The samples were then classified into the diabetic grey-hair group (D-W), the diabetic black-hair group (D-B), the healthy grey-hair group (H-W), or the healthy black-hair group (H-B). The lipid information obtained was imported from the Ezinfo 3.0 software and then adjusted to OPLS-DA mode. The score plots were then viewed to determine any significant differences among the four groups. To filter the lipid S-plot, a restriction of VIP > 1 was used to obtain lipids with greater influence on the groupings. We next determined the obvious differences among the four groups of samples. To screen the lipids in the score plot, VIP > 1 was used as the restriction condition, which can identify lipids with a greater impact on the grouping. We then imported them back to Progenesis QI V2.0 and set further screening conditions: the maximum multiplicity of difference > 2 and *p* < 0.05, which can identify lipids that meet the above requirements. Finally, the ROC curve was plotted. An AUC value > 0.8 and specificity plus sensitivity > 1.6 were taken as the screening conditions to obtain the potentially different lipids. Specific data on characteristic lipid components were obtained through comparison with the LipidMaps database.

### 4.5. Target Acquisition and Gene Interaction Network Construction

The target lipids were searched for relevant genes using pharmmapper (http://www.lilab-ecust.cn/pharmmapper/, accessed on 10 November 2023), a platform for pharmacophore matching and the potential identification of targets. We searched for corresponding protein targets for the matched genes in the UniProt protein database (https://www.uniprot.org/, accessed on 11 November 2023). The screening criteria for UniProt were reviewed. The list of relevant targets obtained from UniProt was imported into the String12.0 web database (https://cn.string-db.org/, accessed on 11 November 2023), and the biological species was limited to “Homo sapiens”. The minimum interaction threshold was set to “highest confidence > 0.9”, the nodes with network breaks were hidden, and the rest were set by default. The data were visualised using the Cytoscape 3.7.1 software to obtain a gene interaction network graph.

### 4.6. Matching Targets to Ingredients and Herbs

The targets were entered into HERB (http://herb.ac.cn/, accessed on 10 November 2023), a database for herbal group identification. Chinese medicines and the active ingredients of those medicines that corresponded to the targets were obtained using the website prediction model. The corresponding lipids/targets/active ingredients/herbal medicines were sorted, classified, and entered into Cytoscape 3.7.1 for visualisation. Correlation networks were constructed, and degree values (degree) were calculated. High-value nodes are often considered to be hubs in a network and may correspond to key biomolecules.

### 4.7. Target KEGG Pathway Enrichment Analysis

The list of genes obtained from pharmmapper was imported into the functional annotation bioinformatics analysis platform database Metascape (https://metascape.org, accessed on 15 November 2023) and personalised by limiting the analysed species to humans (“*H. sapiens*”) and “Custom Analysis”. KEGG pathway enrichment analysis was performed to export the analysis results.

### 4.8. Molecular Docking

The genes in the gene interaction network and the KEGG pathway–gene network were ranked in descending order of degree, and the names of the top 9 genes were intersected to obtain important genes affecting the occurrence of grey hair. The proteins regulated by the important genes were referred to as important protein targets. The 3D structure was obtained from the RCSB PDB protein structure database (https://www.rcsb.org/, accessed on 20 November 2023) and saved in pdb format. The degree values of the active ingredients in the “lipid–target–active ingredient” network were ranked in ascending order, and the top 3 active ingredients were regarded as important active ingredients. The compound structures of the important active ingredients were obtained from PubChem (https://pubchem.ncbi.nlm.nih.gov/, accessed on 21 November 2023), an organic small-molecule bioactivity database. Compound structure files in sdf format were converted into pdbqt format using the open babel 3.1.1 software. Important protein targets were saved in the pdbqt format after removing water molecules and excess ions and adding hydrogen ions using the AutoDockTools 4 software. Molecular docking of important protein targets and important active ingredients was performed using AutoDockTools 4 to obtain molecular docking binding energy scores. The lower the binding energy, the more stable the binding and the stronger the affinity. Finally, the molecular docking results were visualised using the PyMOL 2.5.5 software.

## 5. Conclusions

(1) After analysing the differential lipids of black and white hair in the type 2 diabetes group, black and white hair in the healthy group, and white hair in the diabetes group versus white hair in the healthy group, we concluded that 1-O-alpha-D-glucopyranosyl-1,2-eicosandiol, emmotin A, odyssic acid, PI-Cer, and NAPE might be associated with the production of grey hair in diabetic patients. We also concluded that PS (O-16:0/13:0), PA (12:0/16:1 (9Z)), PS (13:0/20:3 (8Z,11Z,14Z)), GlcCer (d18:1/24:1 (15Z)), and PS (O-20:0/17:2 (9Z,12Z)) might be related to the healthy population’s black hair production; that fucoxanthinol 3-heptadecanoate 3′-myristate and 2-(3-hydroxyphytanyl)-3-phytanyl-sn-glycerol might be associated with the production of grey hair in healthy populations; and that the effects of different lipids on the production of black and grey hair in diabetic and healthy populations are different. This has implications for hair care and product development for different hair types.

(2) Both emmotin A and fucoxanthinol 3-heptadecanoate 3′-myristate are PRs, and their contents in white hair were significantly higher than those of other lipids in the same group. Therefore, we speculate that PRs are related to the production of two types of white hair. This provides a basis for preserving white hair in these two types of women.

(3) Two kinds of PR lipids were analysed via bioinformatics technology and reverse network pharmacology, and 308 targets were matched. Gene interaction network analysis and KEGG pathway enrichment analysis were performed on the 308 targets. The important genes obtained from the two analyses were intersected to obtain three important genes: *PIK3R1*, *AKT1*, and *MAPK1*. A total of 988 ingredients related to nine targets with a moderate value greater than the median of the gene interaction network were screened. The important ingredients (resveratrol, calycosin, and epigallocatechin 3-gallate) and important herbs (glossy privet fruit, etc.) were obtained through a network diagram. Ingredients and herbs that may have inhibitory effects on the occurrence of white hair can provide a basis and direction for the development of anti-white-hair raw materials. The specific efficacy of these ingredients and herbs needs to be further verified with experiments.

## Figures and Tables

**Table 1:** Lipids with significant differences between D-B and D-W.

NO.	Compound ID	Description	AUC	ANOVA (*p*)	Fold Change	Sens. + Spec.	Highest
1	LMFA13010005	1-O-alpha-D-2- glucopyranosyl-1,2-eicosandiol	0.929	0.000361386	2.252063817	1.826	DW
2	LMFA01031114	Odyssic acid	0.893	0.000142586	3.055375064	1.727	DW
3	LMPR0103200001	Emmotin A	0.894	0.000080271	3.903529404	1.728	DW
4	LMSP05010078	GlcCer(t16:0(15Me)/23:0(2OH[R]))	0.124	0.005676188	2.086677539	1	DB
5	LMPK12010247	Alatanin 2	0.792	0.002986347	2.401225242	1.478	DW
6	LMGP02010358	NAPE(18:1(9Z)/16:1(9Z)/18:0)	0.873	0.004469992	2.229707111	1.654	DW
7	LMPR01070357	Fucoxanthinol 3-heptadecanoate 3′-myristate	0.079	3.936789783	3.465273379	1	DB
8	LMSP03030041	PI-Cer(t18:0/26:0(2OH))	0.827	0.003129991	2.331419075	1.628	DW
9	LMGP03020002	PS(O-16:0/13:0)	0.693	0.006619901	2.295211161	1.309	DW
10	LMGP03020024	PS(O-18:0/17:0)	0.553	0.041367203	2.389873728	1.195	DW

**Table 2:** Lipids with significant differences between H-B and H-W.

NO.	Compound ID	Description	AUC	ANOVA (*p*)	Fold Change	Sens. + Spec.	Highest
1	LMFA08010008	Linoleamide	0.095	0.000117489	2.276516518	1	HW
2	LMSP02020022	Cer(d18:0/17:0)	0.121	0.000157643	2.353057800	1.096	HW
3	LMPK12010431	6-Hydroxydelphinidin3-glucoside	0.118	1.70 × 10^−11^	6.142355572	1.004	HW
4	LMFA13030011	3-O-alpha-L- rhamnopyranosyl-3- hydroxynonanoyl-3-hydroxydecanoic acid	0.056	7.58 × 10^−7^	4.414402260	1	HW
5	LMGL02010258	DG(22:0/22:0/0:0)	0.142	0.022482587	2.169901443	1	HW
6	LMGP10010047	PA(12:0/16:1(9Z))	0.891	0.010546977	2.624807539	1.816	HB
7	LMGP03010085	PS(13:0/20:3(8Z,11Z,14Z))	0.882	0.008551482	2.604751342	1.815	HB
8	LMFA07090116	FAHFA(3:0/2-O-24:0)	0.087	9.99 × 10^−16^	2.137504697	1.034	HW
9	LMGL02010226	DG(20:5(5Z,8Z,11Z,14Z,17Z)/20:5(5Z,8Z,11Z,14Z,17Z)/0:0)	0.103	0.014134289	2.013484367	1.034	HW
10	LMSP0501AA08	GlcCer(d18:1/24:1(15Z))	0.858	4.49 × 10^−5^	2.163027520	1.816	HB
11	LMFA07050135	(9Z)-myristoleoyl-CoA	0.276	0.000491142	16.73757043	1	HW
12	LMGP03020002	PS(O-16:0/13:0)	0.904	9.37 × 10^−6^	2.041614980	1.85	HB
13	LMGP03020050	PS(O-20:0/17:2(9Z,12Z))	0.88	0.032153449	7.412690677	1.728	HB

**Table 3:** Lipids with significant differences between D-W and H-W.

NO.	Compound ID	Description	AUC	ANOVA (*p*)	Fold Change	Sens. + Spec.	Highest
1	LMFA13010005	1-O-alpha-D-glucopyranosyl-1,2-eicosandiol	0.032	4.44 × 10^−15^	3.742098018	1	DW
2	LMSP01040001	C16 sphinganine	0.072	0.000124182	2.252825814	1	DW
3	LMPR0103200001	Emmotin A	0.27	0.018592574	2.190404243	1	DW
4	LMPK12010247	Alatanin 2	0.219	0.005018743	2.019533277	1	DW
5	LMPK12010178	Cyanidin 3-(6″-sinapylsophoroside)-5- glucoside	0.111	8.63 × 10^−10^	2.211321943	1	DW
6	LMFA07070055	Cervonyl carnitine	0.043	2.60 × 10^−11^	2.200801464	1	DW
7	LMGP02010358	NAPE(18:1(9Z)/16:1(9Z)/18:0)	0.125	0.00021046	2.014682746	1.024	DW
8	LMPR01070357	Fucoxanthinol 3-heptadecanoate 3′-myristate	0.917	3.98 × 10^−6^	2.664853108	1.772	HW
9	LMGL02030034	2-(3-Hydroxyphytanyl)-3-phytanyl-sn-glycerol	0.833	2.93 × 10^−7^	2.133485786	1.634	HW
10	LMGP06010306	PI(18:1(9Z)/22:2(13Z,16Z))	0.037	1.00 × 10^−13^	2.050766857	1	DW
11	LMGP03010243	PS(17:0/21:0)	0.033	3.60 × 10^−5^	4.607339045	1	DW
12	LMGP03020002	PS(O-16:0/13:0)	0.025	4.16 × 10^−13^	5.911351804	1	DW

**Table 4:** Top nine ingredients in terms of degree value.

NO.	Ingredient ID	Ingredient Name	Degree
1	HBIN042111	Resveratrol	13
2	HBIN019425	Calycosin	13
3	HBIN025347	Epigallocatechin 3-gallate	12
4	HBIN016408	Apigenin	11
5	HBIN029531	Honokiol	11
6	HBIN047613	Ursolic acid	10
7	HBIN039998	Pinocembrin	9
8	HBIN021460	Cordycepin	9
9	HBIN024134	Dioscin	9

**Table 5:** Top nine herbs in terms of degree value.

NO.	Herb ID	Chinese Name	Degree
1	HERB004242	Fruit of glossy privet	3
2	HERB001779	Root of Ural liquorice	3
3	HERB001901	Folium ilicis cornutae	3
4	HERB004327	Fructus paulowniae fortunei	2
5	HERB003252	Bluesepal rabdosia	2
6	HERB002983	All grass of fineleaf schizonepeta	2
7	HERB004470	Spiked loosestrife equivalent plant: *Lythrum anceps*	2
8	HERB003636	European verbe herb	2
9	HERB003658	Ephedra	2

**Table 6:** Biological pathways associated with hair growth or melanin synthesis.

Biological Pathway	Literature Validation	Literature
PI3K-Akt signalling pathway	The use of PI3K/Akt inhibitors or activators can reduce or increase the hair growth induction ability of dermal papilla cells. Pyruvate and ethyl pyruvate inhibit melanogenesis through PI3K/Akt and GSK3β signal transduction and by targeting the ERK and GSK3β pathways, respectively.	[18,19]
MAPK signalling pathway	During the proliferation and differentiation of melanocytes and melanin synthesis, the p38MAPK signalling pathway regulates melanin synthesis by acting on MITF.	[14]
FoxO signalling pathway	FoxO 6 is an antioxidant gene that prevents oxidative-stress-induced melanogenesis.	[20]
PPAR signalling pathway	RNA isolated from the scalp adipose tissue of men with androgenetic alopecia was sequenced, and differential gene expression and pathway analysis were performed. The results showed that the PPAR signalling pathway of the bald scalps was the most significantly downregulated.	[21]
VEGF signalling pathway	VEGFR-2 (VEGF receptor 2) is expressed in primary human dermal papilla cells, and VEGF induces the proliferation of human dermal papilla cells through the VEGFR-2/ERK pathway.	[22]
Neurotrophin signalling pathway	Neurotrophin (NT)-4 is an inducing factor in the degenerative period of the hair growth cycle. Androgen-promoting NT-4 activity may be related to the pathogenesis of AGA.	[23]
Melanoma	——	—
HIF-1 signalling pathway	The downregulation of HIF-1 pathway-related genes (EGLN 1 and EGLN 3) can promote hair follicle growth.	[24]
Sphingolipid signalling pathway	Ceramide is a biologically active sphingolipid that reduces melanin synthesis in Mel-Ab cells.	[25]
Steroid hormone biosynthesis	Androgen-mediated androgenetic alopecia (AGA) is the thinning and shedding of hairs due to a shortened anagen phase in the hair cycle and follicular atrophy, a process that is also influenced by 5 alpha-reductase and testosterone, among other things.	[26]
AMPK signalling pathway	Lipocalin mediated through the AMPK activator (AICAR) can downregulate the expression of MITF, tyrosinase, TRP-1, and DCT and reduce melanin content in normal human and mouse melanocytes.	[27]
cAMP signalling pathway	The promoter region of the MITF gene binds to the phosphorylated cAMP response element-binding protein (p-CREB), promoting the expression of associated proteins and the MITF gene. In turn, this increases the expression of the TYR gene family of proteins and, finally, promotes melanin synthesis.	[14]
NF-kappa B signalling pathway	The tyrosinase inhibitor MHY884 inhibits the UVB-induced activation of the NF-kB signalling pathway by downregulating oxidative stress.	[28]
Tyrosine metabolism	Tyrosinase (TYR) is a key rate-limiting enzyme in melanin synthesis; the TYR-related proteins TRP-1 and TRP-2 play equally important roles in melanin synthesis.	[14]
Melanogenesis	——	—

**Table 7:** Molecular docking binding energy scores (kcal/mol).

Ingredient Gene Name	*PIK3R1*	*AKT1*	*MAPK1*
Resveratrol	−5.01	−3.78	−4.75
Calycosin	−5.49	−4.42	−4.12
Epigallocatechin 3-gallate	−3.24	−1.42	−2.45

## Data Availability

The data presented in this study are available upon request from the corresponding author due to privacy concerns.

## Abbreviations

D	Volunteers with type 2 diabetes
H	Healthy volunteers
D-W	White hair in group D
D-B	Black hair in group D
H-W	White hair in group H
H-B	Black hair in group H
UPLC-QTOF-MS	Ultra-HPLC-QTOF-MS (Ultra-Performance Liquid Chromatography–Tandem Time-of-Flight Mass Spectrometry)
OPLS-DA	Orthogonal Partial Least Squares Discriminant Analysis
ROC	Receiver operating characteristic
AUC	Area under the curve
FA	Fatty acid
PR	Pregnenolone lipid
GP	Glycerophospholipid
GL	Glycerolipid
SP	Sphingolipid
